# Case Report: From idiopathic recurrent pericarditis to systemic Behçet’s Disease: unmasking a unified IL-1-driven autoinflammatory phenotype

**DOI:** 10.3389/fimmu.2026.1900526

**Published:** 2026-08-14

**Authors:** Bizzi Emanuele, Mauro Angela, Cristiano Germinario, Casarin Francesca, Berra Silvia, Ministrini Stefano, Gidaro Antonio, Manzotti Giuseppina, Marra Alessandro Maria, Rotunno Sara, Greco Elisabetta, Modica Stella, Paciullo Francesco, Sculco Martina, Damanti Sarah, Abatianni Giulia, Gagliardi Caterina, Rovere Querini Patrizia, Brucato Antonio

**Affiliations:** 1 Department of Internal Medicine, Fatebenefratelli Hospital, Azienda Socio-Sanitaria Territoriale (ASST) Fatebenefratelli and Sacco Hospitals, Milan, Italy; 2 Department of Pediatrics, Fatebenefratelli Hospital, Milan, Italy; 3 Farmacia Marsico, Acquaviva Delle Fonti, Bari, Italy; 4 Center for Molecular Cardiology, University of Zurich, Zurich, Switzerland; 5 Department of Internal Medicine, Sacco Hospital, Azienda Socio-Sanitaria Territoriale (ASST) Fatebenefratelli and Sacco Hospitals, Milan, Italy; 6 Allergology Service, Casa di Cura Beato Palazzolo, Bergamo, Italy; 7 Pulmonology Unit, Azienda Socio-Sanitaria Territoriale (ASST) Rhodense, Garbagnate Milanese Hospital, Milan, Italy; 8 Internal Medicine, S.Pietro Fatebenefratelli Hospital, Rome, Italy; 9 Rheumatology, Allergology and Clinical Immunology, Department of “Medicina dei Sistemi”, University of Rome Tor Vergata, Rome, Italy; 10 Department of Internal Medicine, Vita-Salute San Raffaele Hospital, Milan, Italy

**Keywords:** Anakinra, autoinflammation, Behçet’s Disease, cardio-Behçet, IL-1 blockade, NLRP3 inflammasome, personalized therapeutics, recurrent pericarditis

## Abstract

**Background:**

Recurrent idiopathic recurrent pericarditis (RP) is increasingly recognized as an organ-specific autoinflammatory syndrome driven by the interleukin-1 (IL-1) axis. Behçet’s Disease (BD), a systemic vasculitis, exhibits significant pathogenetic overlap with RP through IL-1 mediated hyperinflammation, particularly in phenotypes dominated by serositis. The clinical evolution of prolonged, seemingly idiopathic RP into overt, systemic BD upon the tapering of targeted therapy remains a critical, yet underreported, observation.

**Case presentation:**

A 42 years old female with corticosteroid-dependent, colchicine-resistant RP achieved complete and sustained clinical and biochemical remission using the IL-1 receptor antagonist, Anakinra, for almost 30 months, despite the tapering started after 18 months of continuous therapy with Anakinra, associated to colchicine and a quick tapering and withdrawal of corticosteroids. Following the elective tapering of Anakinra, the patient remained stable for three months with a dose of an injection of 100mg of Anakinra three times per week, before suffering a severe pericardial relapse concurrent with the systemic onset of systemic BD. The clinical condition observed fulfilled the International Criteria for Behçet’s Disease (ICBD), featuring recurrent oral and genital ulcerations, erythema nodosum and a positive pathergy test. The immediate reinitiation of Anakinra (100mg daily) led to the rapid and complete resolution of both the pericardial inflammation and all systemic mucocutaneous manifestations.

**Conclusions:**

This case highlights a potential pathogenetic overlap between refractory RP and serositis-dominant BD phenotypes, suggesting that isolated RP may, in select cases, represent an early mono-organ precursor of a systemic autoinflammatory propensity. Furthermore, it provides clinical rationale for considering IL-1 blockade in serositis-dominant BD, though larger prospective studies are needed to confirm these findings.

## Introduction

Behçet’s Disease (BD) is a multifaceted systemic inflammatory disorder classically characterized by recurrent oral ulcers, genital ulcers, and uveitis, but its clinical spectrum encompasses a wide range of mucocutaneous, ocular, vascular, neurological, and musculoskeletal manifestations (1–6). While historically categorized as a systemic vasculitis, contemporary immunological insights view BD as a bridge between autoimmunity and autoinflammation (7, 8). At the molecular level, its pathogenesis involves an intricate interaction between genetic factors, notably the HLA-B51 allele, and environmental triggers that spark inflammasome activation and the overproduction of proinflammatory cytokines, particularly interleukin-1 (IL-1) and TNF-alpha (8, 9). Managing refractory BD phenotypes remains a significant clinical challenge (10). Pericardial involvement represents a less common manifestation, but emerging evidence suggests that certain mono-organ presentations may actually serve as the initial phase of a broader systemic autoinflammatory propensity, which fully declares itself only upon the tapering of targeted immunosuppression (1, 6).

In parallel, recurrent pericarditis (RP) is a debilitating autoinflammatory condition characterized by repeated flares of pericardial inflammation. Once labeled as idiopathic, contemporary molecular research has redefined a major subset of RP as a prototypical organ-specific autoinflammatory syndrome (11–16). This paradigm shift relies on the pivotal role played by the NLRP3 inflammasome (17–23), which processes and releases bioactive IL-1beta in response to cellular danger signals (17, 18). Dysregulation of this axis leads to a self-perpetuating inflammatory loop refractory to conventional therapies such as NSAIDs and colchicine, often resulting in corticosteroid dependence (24–31). The clinical success of targeted IL-1 blockers like Anakinra and Rilonacept has not only transformed RP management but has also acted as a diagnostic *ex juvantibus* confirmation of its autoinflammatory nature.

The diagnostic boundaries between isolated RP and systemic vasculitides like BD are increasingly blurred, as both conditions share a reliance on the IL-1 axis, particularly in cases where serositis is a dominant feature (6, 9, 10, 32–38). Cardiac involvement in BD, termed cardio-Behçet, occurs in 1% to 6% of patients (36–43). Pericarditis is among the most frequent cardiac findings, often presenting in association with other cardiac lesions or as part of a broader autoinflammatory cluster involving serosal, mucocutaneous, and articular manifestations (11, 12, 44–46). Crucially, pericarditis can act as the inaugural or sentinel sign of BD, preceding classic diagnostic features by months or years (47–55).

In these scenarios, extensive clinical series highlight that diagnostic latency often leads to an initial classification of idiopathic RP (49–52, 54–60). Pericarditis can occur concurrently with severe vascular or intracardiac complications, including intracardiac thrombi, valvular sequelae, aortic aneurysms, or coronary involvement (48, 61–63). Recognizing specific autoinflammatory clusters driven by innate immunity activation is therefore essential for guiding personalized therapy (34–40, 43, 64, 65). Recent evidence underlines that this autoinflammatory phenotype is primarily driven by IL-1 dysregulation, explaining why serositis-dominant presentations show a distinct therapeutic response to IL-1 antagonists (66).

The molecular connection between RP and BD lies in shared innate immune pathways (6–8, 32, 67–69), where NLRP3 assembly and IL-1beta release orchestrate localized or systemic hyperinflammation (33, 70–74). In RP, danger signals trigger localized pericardial neutrophil recruitment (29–31, 75–77), whereas in BD, systemic NLRP3 overexpression drives widespread tissue involvement (6, 7, 32, 78). While international consensus and ESC guidelines provide structured protocols for biological dose reduction (16, 79), tapering IL-1 blockade can unmask latent systemic drive. Here, we present a case where elective Anakinra tapering unmasked systemic BD in a patient long managed for refractory RP, illustrating the shared autoinflammatory spectrum underlying these conditions.

## Case presentation

Patient information: a 42 years old caucasian female presented to our immunology clinic with a longstanding history of RP. Her medical history was otherwise unremarkable, with no family history of autoimmune or autoinflammatory diseases. Prior to the current episode, she had experienced multiple flares of pericarditis (9 episodes) over a seven years period, characterized by pleuritic chest pain, pericardial friction rubs and diffuse ST-segment elevation on electrocardiogram (ECG). Most of the previous episodes of RP had occurred when she had attempted tapering of prednisone below 15 mg/day, while maintaining the background therapy of indomethacin 50 mg three times daily and colchicine 0.5 mg daily. During the initial 7-year pre-referral period, the patient was managed at primary/secondary centers for isolated recurrent pericarditis. Systemic autoinflammatory screening and pathergy testing were not performed during those early flares, and while no major extra-cardiac events were reported, the presence of minor, unrecorded mucocutaneous symptoms during that period cannot be definitively ruled out. Upon referral to our immunology clinic (Month 1), a detailed systemic review was negative for ocular, vascular, articular, or persistent mucocutaneous lesions; however, formal pathergy testing was deferred until the acute multi-system flare at Month 33.

## Clinical findings

At the time of her initial referral to our center, the patient was corticosteroid dependent (requiring 15mg/day of prednisone to suppress symptoms) and colchicine resistant, and presented a new RP flare after stopping NSAID therapy for approximately one week, without tapering NSAID dose. Physical examination typically revealed slight tachycardia and muffled heart sounds, with laboratory findings showing markedly elevated C-reactive protein (CRP) (85 mg/L) and an increased white blood cell count with marked neutrophilia, while extensive infectious (blood cultures for aerobic and anaerobic bacteria, Interferon-Gamma Release Assay for *Mycobacterium tuberculosis*, HIV-1/2 serology, hepatitis B and C serology and serology for cardiotropic viruses such as Coxsackievirus, Echovirus, Adenovirus, Parvovirus B19, EBV, CMV, HSV, *Borrelia burgdorferi*, *Coxiella burnetii*, *Mycoplasma pneumoniae*, Syphilis screening), neoplastic (chest X-ray and complete abdominal ultrasound examination) and autoimmune workups (including ANA, ANCA and ENA panels) were consistently negative, leading to a diagnosis of idiopathic recurrent pericarditis.

Comprehensive biochemical evaluation confirmed normal renal function (serum creatinine 0.72 mg/dL, estimated glomerular filtration rate >90 mL/min/1.73 m²) and normal urinalysis without microscopic hematuria, active urinary sediments, or proteinuria (24-hour urine protein <0.15 g/24h), ruling out renal involvement or secondary amyloidosis. Pericardiocentesis was withheld as the effusion remained non-tamponading (30 mm max, without echocardiographic signs of hemodynamic compromise) in accordance with current guidelines. However, the presence of concurrent pleural and peritoneal effusions during acute inflammatory flares was consistent with an autoinflammatory exudative polyserositis phenotype.

To rule out underlying monogenic autoinflammatory syndromes, Next-Generation Sequencing (NGS) panel screening for targeted genes (including MEFV, TNFRSF1A, NLRP3, MVK, and PSTPIP1) was performed during the initial evaluation of refractory pericarditis, showing no pathogenic or likely pathogenic variants.

Along with ultrasound confirmation of pericarditis, which showed thickened pericardial sheets and non-tamponading pericardial effusion approximately 20mm thick, the patient also showed signs of minimal pleural and peritoneal effusion.

## Therapeutic interventions (Phase I)

Given the refractory nature of her condition and the side effects of chronic steroid use, treatment with the IL-1 receptor antagonist Anakinra (100mg daily, subcutaneously) was initiated. The clinical response was immediate and dramatic: CRP levels normalized within two weeks and she achieved complete symptomatic remission in less than a week; over the following 30 months, despite the slow tapering of Anakinra, she remained asymptomatic, allowing for the successful tapering and discontinuation of corticosteroids, while colchicine therapy (0,5mg/day) was still ongoing.

## Timeline and the systemic event

After 18 months of sustained daily administration of Anakinra and clinical stability, followed by a slow tapering performed by interruption of one of the weekly doses every 3 months (6 times/week for three months, then 5 times/week for another 3 months, then 4 times/week for three months, then 3 times/week for three months) a slight increase of CRP was observed after two months of Anakinra administration 3 times a week (8mg/L) without clinical consequences, and the administration of Anakinra was stabilized to three times a week. However, in the third month, she suffered a systemic relapse.

## Diagnostic assessment

Upon arrival at the Emergency Department, the patient presented with acute, severe retrosternal chest pain and dyspnea. Simultaneously, she exhibited multiple painful oral aphthous ulcers consisting of more than three lesions, deep scarred genital ulcerations and tender erythematous nodules on her lower shins, clinically consistent with erythema nodosum. A pathergy test performed via needle prick also yielded a positive result within 24 hours. Transthoracic echocardiography showed a 30mm pericardial effusion without signs of incipient tamponade, while laboratory investigations revealed a significant CRP spike of 120 mg/L. Genetic testing for HLA-B51 was performed during the acute multi-system flare and returned negative. While HLA-B51 is a recognized genetic susceptibility marker for BD, its absence does not preclude the diagnosis, particularly in non-ocular, serositis-dominant phenotypes. Based on the concurrent presence of oral and genital ulcers, erythema nodosum and the positive pathergy test, the patient achieved a score of 7 points on the International Criteria for Behçet’s Disease (ICBD), with this total far exceeding the diagnostic threshold of 4, effectively confirming a transition from seemingly isolated recurrent pericarditis to systemic Behçet’s Disease.

## Therapeutic interventions (Phase II) and outcomes

**Figure 1:**
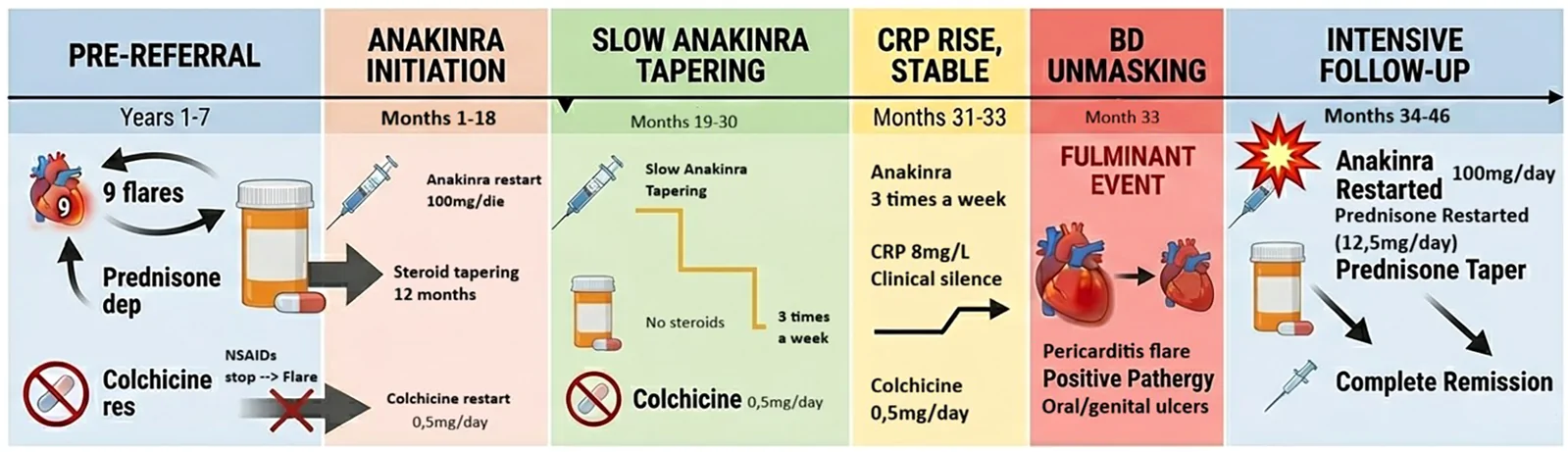
Detailed disease course. During the pre-referral period (Years 1–7), the patient experienced 9 recurrent pericarditis flares. These episodes were characterized by corticosteroid dependence and colchicine resistance. A severe flare occurred following NSAID cessation. Upon referral (Months 1–18), daily Anakinra (100 mg) was initiated. This led to immediate clinical and biochemical remission. Corticosteroids were tapered over 12 months. During the Anakinra tapering phase (Months 19–30), the Anakinra dose was gradually reduced. Background colchicine therapy (0.5 mg/day) remained ongoing. At three injections per week (Months 31–33), a slight asymptomatic CRP rise (8 mg/L) was noted. A systemic relapse occurred at Month 33. This event unmasked systemic Behçet’s Disease, including a 30 mm pericardial effusion, oral/genital ulcers and a positive pathergy test. The patient achieved an ICBD score of 7. Intensive follow-up (Months 34–46) involved restarting daily Anakinra and 12.5 mg/day of prednisone. Complete remission was maintained as prednisone was tapered to 5 mg/day by month 12 of follow-up. BD, Behçet’s Disease; CRP, C-reactive protein; ICBD, International Criteria for Behçet’s Disease; IL-1, Interleukin-1; NSAIDs, Non-steroidal anti-inflammatory drugs; RP, Recurrent pericarditis.

After a telephone consultation regarding the case, and also in accordance with the patient’s decision, instead of increasing the dosage of prednisone, it was decided to restart therapy with Anakinra at 100 mg/day with one administration per day. The clinical response was swift, with the patient’s chest pain gradually and completely resolving within 72 hours. Within one week, the mucocutaneous lesions showed significant healing and a followup echocardiography performed at 10 days confirmed the substantial resolution of the pericardial effusion (20mm). Following this intervention, the patient has remained in complete clinical and biochemical remission for over 12 months on continuous Anakinra therapy, with no further flares reported to date. A subsequent cardiac MRI, performed 1 month after Anakinra full dose restart, revealed a condition of chronic pericarditis, with thickening of the pericardial layers, without the characteristics of constrictive pericarditis and without further signs of cardiac involvement typical of BD, while a repeat of the same examination after 9 months highlighted a stable cardiac and pericardial condition with only a reduction in the effusion, detectable only posteriorly and less than 10 mm thick. A cautious tapering of the prednisone dosage was started 3 months after the clinical condition stabilized, mainly at the patient’s request, reaching 12 months of followup with a maintenance dose of approximately 5 mg of prednisone per day (**Figure 1**).

## Patient perspective

The patient reported a significant improvement in her quality of life following the definitive diagnosis and the subsequent stabilization of her therapy. She emphasized that while the long standing recurrent pericarditis had been physically and mentally exhausting, the sudden onset of systemic mucocutaneous symptoms was particularly distressing. The rapid resolution of both the cardiac and systemic manifestations upon the reinitiation of full dose of IL-1 blockade provided the patient with a renewed sense of security and she expressed high treatment satisfaction and a strong commitment to long term adherence to the daily subcutaneous injection regimen. Furthermore, given her stable clinical status and significant concerns regarding potential long term side effects, the patient strongly requested a tapering of her prednisone dosage.

## Literature review and discussion

Pericardial involvement in BD is a recognized, albeit relatively infrequent, manifestation of the cardio-Behçet spectrum (49–52, 54–60). Large scale cohort studies and systemic reviews have estimated the prevalence of cardiac involvement to range between 1% and 6% of patients, with significant geographical variations, often appearing more frequently in cohorts from the Mediterranean basin and the Middle East. Among these cardiac manifestations, pericarditis is frequently identified as the most common clinical finding, often occurring in association with other cardiac lesions such as intracardiac thrombi, endomyocardial fibrosis or coronary vasculitis (48, 61–63). A critical observation in the current literature is the temporal relationship between pericardial inflammation and the full expression of BD. While pericarditis often occurs during the established course of the disease, it has been increasingly documented as a sentinel sign or the inaugural manifestation of the syndrome. In several reported cases, RP preceded the diagnostic mucocutaneous or ocular symptoms by several months or even years (51–54, 56). This pre-vasculitic phase often leads to an initial diagnosis of idiopathic recurrent pericarditis, delaying the identification of the underlying systemic pathology until a major flare or the withdrawal of non-specific immunosuppression unmasks the multiorgan involvement.

## Clinical clustering and the autoinflammatory phenotype

Recent phenotypic analyses have increasingly focused on the necessity of categorizing BD into specific clinical clusters to better facilitate a more nuanced and guided approach to personalized therapy (34–40, 43, 64, 65). According to pivotal studies, such as those conducted by Emmi et al., a distinct and well-defined autoinflammatory cluster has emerged within the broader spectrum of the disease. This particular phenotype is primarily characterized by the presence of recurrent serositis, which frequently manifests as pericarditis, pleuritis, or a combination of both (66).

This clinical subset often presents with significant mucocutaneous involvement, specifically involving papulopustular skin lesions and persistent acneiform eruptions that distinguish it from the more traditional vasculitic forms of the disorder, and beyond these features, patients within this cluster commonly experience systemic articular involvement, adding a musculoskeletal dimension to their inflammatory profile. This specific pedigreed autoinflammatory endotype is notably driven by dysregulation within the IL-1 pathway rather than traditional vasculitic triggers, often resulting in a clinical course marked by high recurrence rates and significant corticosteroid dependence, yet showing a lower incidence of major macrovascular events like deep vein thrombosis. Identifying this cluster is of paramount importance for the clinician, as these patients typically exhibit an exceptional and rapid therapeutic response to IL-1 receptor antagonists, reinforcing the idea that in cases of refractory recurrent pericarditis, a meticulous and exhaustive search for these subtle clinical markers is mandatory for accurate disease classification.

The transition from organ specific pericardial inflammation to systemic vasculitis, as observed in our case, may find its molecular explanation in the shared activation of the innate immune system (6–8, 32, 67–69). Both RP and BD are now recognized as being driven by the IL-1 pathway, with the NLRP3 inflammasome acting as the key intracellular sensor and orchestrator of the inflammatory response (33, 70–74).

The systemic onset observed in our patient following the tapering of Anakinra serves to highlight several critical immunological phenomena that are essential for understanding the management of autoinflammatory diseases. Primarily, this case underscores the vital distinction between clinical suppression and true disease resolution. While IL-1 blockade is remarkably effective at silencing overt clinical manifestations, it does not necessarily eliminate the underlying genetic or epigenetic predispositions toward inflammasome overactivation. Consequently, these latent pathogenic mechanisms can be rapidly reactivated once the protective shield of the IL-1 receptor antagonist is reduced.

While the individual pathogenic roles of IL-1 in both RP and BD are well recognized, this case offers several distinct insights that bridge the gap between organ-specific autoinflammation and systemic vasculitis. In contrast to typical presentations of cardio-Behçet, where pericardial involvement occurs concurrently with or secondary to systemic manifestations, our patient exhibited a 7-year history of isolated, corticosteroid-dependent RP. This prolonged diagnostic latency suggests that what is initially classified as idiopathic RP may, in a subset of patients, represent a prolonged mono-organ precursor or forme fruste of systemic BD. Furthermore, a key novel observation of this report lies in the pharmacological dynamic associated with targeted IL-1 inhibition. Continuous treatment with Anakinra provided profound suppression of inflammation, maintaining complete clinical stability for nearly 30 months; however, its elective tapering triggered a rapid, systemic flare that unmasked the latent multi-system BD phenotype. This critical event highlights that while IL-1 blockade effectively silences systemic hyperinflammation, it may not eradicate the underlying pathogenic drive, meaning that dose reduction can lower the therapeutic threshold and expose previously silent systemic features. In this context, the case provides direct clinical and diagnostic ex juvantibus validation for recent phenotypic classifications of BD that delineate an autoinflammatory cluster characterized by serositis, articular involvement, and prominent mucocutaneous lesions. The rapid and synchronized clearance of severe pericardial effusion, bipolar ulcerations, and erythema nodosum upon full-dose Anakinra re-challenge confirms that this specific BD endotype is driven predominantly by the IL-1 axis, effectively distinguishing it from traditional vasculitic phenotypes that typically require TNF-alpha targeted or cytotoxic therapies. Ultimately, these findings carry practical algorithmic value for the clinical management of refractory pericarditis: in patients undergoing IL-1 receptor antagonist tapering, clinicians should maintain high vigilance for emerging multi-system autoinflammatory signs, utilizing targeted screening—such as pathergy testing and meticulous mucocutaneous inspection—to guide disease re-classification and personalized biological maintenance strategies.

## Personalized therapeutics: beyond TNF inhibition

While TNFalpha inhibitors have traditionally been the mainstay of biological therapy in BD, they are not always effective for the serositic phenotype (33, 70–74). The literature increasingly supports IL-1 antagonism as a personalized therapeutic strategy for the serositis cluster of BD. For patients presenting with refractory RP, the use of Anakinra not only treats the immediate cardiac threat but may also serve as a targeted prophylaxis against the systemic evolution of the disease.

While the patient met the clinical criteria for BD (ICBD score of 7), it is important to acknowledge the phenotypic overlap between BD and polygenic or monogenic autoinflammatory diseases. Symptoms such as recurrent serositis, bipolar ulceration, and a robust response to IL-1 blockade are hallmark features of innate immune system hyperactivation. The absence of pathogenic variants on NGS autoinflammatory panel screening supports a clinical diagnosis of serositis-dominant BD rather than a classical monogenic periodic fever syndrome, though it reinforces the concept of BD as a disease bridging autoimmunity and autoinflammation.

Although the rapid response to Anakinra observed in this patient aligns with the hypothesis of an IL-1-driven autoinflammatory phenotype, therapeutic recommendations cannot be generalized from a single case observation. While IL-1 blockade offers a promising pathophysiologically targeted option for patients presenting with predominant serositis, robust confirmation in larger prospective cohorts and controlled clinical trials is strictly required before anti-IL-1 agents can be formally incorporated into preferential treatment algorithms for BD.

The clinical evolution of this case underscores the necessity of moving toward a more personalized, pathophysiology driven approach in the management of refractory serositis. While RP is frequently managed as an isolated cardiac phenomenon, our findings suggest that in a subset of patients, it represents the localized expression of a systemic autoinflammatory propensity. Therefore, a refined therapeutic algorithm must begin with a high index of clinical suspicion for BD, even when the classic diagnostic criteria are not initially met.

In patients presenting with corticosteroid-dependent and colchicine-resistant RP, the clinician should look beyond the pericardium. A meticulous search for minor or hidden autoinflammatory markers, such as occasional oral aphthosis, atypical acneiform back lesions or a positive pathergy test, is mandatory. This approach, which is not limited to pericardial pathology alone but also contemplates possible systemic involvement in the course of pericarditis, has already been suggested in numerous previous studies, in which, alongside the phenomena of inflammation of the pericardium, involvement of the pleura or peritoneum was observed, leading to the configuration of systemic conditions of polyserositis, on an autoinflammatory basis (Princess Clotilde syndrome) (11). In other Patients, these subtle signs may help distinguish a truly idiopathic pericarditis from a pre-vasculitic or serositis-dominant phenotype of BD and in this context, the rapid response to Anakinra acts as more than just a treatment, it provides useful ex juvantibus clinical information. A normalization of inflammatory markers and resolution of symptoms within 3 days supports the IL-1 dependency of the disease, identifying an endotype that is fundamentally driven by the innate immune system.

However, as demonstrated by the relapse in our patient, achieving clinical remission does not imply the resolution of the underlying immunological drive. Consequently, any tapering protocol must be gradual and accompanied by rigorous systemic surveillance. Unlike the standard vasculitic phenotype of BD, which often necessitates TNF-alfa inhibition for ocular or major vascular involvement, the serositis-dominant cluster appears sensitive to IL-1 antagonism. For these patients, Anakinra could be considered not merely as a second line option for the heart, but as the primary targeted therapy to maintain systemic stability and prevent the transition toward multiorgan vasculitis.

The elective tapering of Anakinra represents one of the most formidable challenges in the management of refractory autoinflammatory syndromes. While international guidelines such as the 2025 ESC (European Society of Cardiology) Guidelines provide structured frameworks and expert-based protocols to direct the reduction of IL-1 blockade (16, 79), the practical application of these algorithms is often complicated by the profound heterogeneity of individual disease phenotypes. The success of a tapering strategy is not merely a matter of pharmacological half-life, but is heavily influenced by the specific autoinflammatory load of the patient and the potential for a systemic rebound effect upon dose reduction. Furthermore, clinical practice must navigate the complex interplay between physician expertise and patient expectations. While clinicians and patients may feel pressured to reduce biological exposure due to long term safety concerns or healthcare costs, patients often harbor significant anxiety regarding the recurrence of debilitating symptoms, which can lead to divergent attitudes toward tapering.

## Limitations

A limitation of this report is the lack of clinical photographs of the acute mucocutaneous lesions; the acute flare was documented during emergency presentation by the attending clinical team who prioritized rapid diagnostic scoring (ICBD score of 7) and immediate therapeutic intervention over photographic archiving.

## Conclusions

This case highlights that long-standing, corticosteroid-dependent recurrent pericarditis may represent an early mono-organ precursor or manifestation of a systemic autoinflammatory phenotype, such as serositis-dominant BD. Clinicians should be aware that the elective tapering of targeted IL-1 blockade can unmask latent systemic features. When reducing IL-1 inhibitors in patients with refractory pericarditis, close multi-system monitoring—including screening for mucocutaneous lesions and pathergy testing—is essential. While re-initiating full-dose IL-1 blockade induced rapid disease control in our patient, prospective cohort studies are required to confirm the broader applicability of IL-1 targeted strategies in this specific clinical subtype.

## Data Availability

The original contributions presented in the study are included in the article/supplementary material. Further inquiries can be directed to the corresponding author.
